# Impacts of Removing Badgers on Localised Counts of Hedgehogs

**DOI:** 10.1371/journal.pone.0095477

**Published:** 2014-04-15

**Authors:** Iain D. Trewby, Richard Young, Robbie A. McDonald, Gavin J. Wilson, John Davison, Neil Walker, Andrew Robertson, C. Patrick Doncaster, Richard J. Delahay

**Affiliations:** 1 National Wildlife Management Centre, Animal Health and Veterinary Laboratories Agency, New Haw, Addlestone, United Kingdom; 2 Durrell Wildlife Conservation Trust, Les Augrès Manor Trinity, Jersey, Channel Islands, United Kingdom; 3 Environment and Sustainability Institute, University of Exeter, Penryn, Cornwall, United Kingdom; 4 Institute of Ecology and Earth Sciences, University of Tartu, Tartu, Estonia; 5 Centre for Biological Sciences, University of Southampton, Southampton, United Kingdom; CNRS, France

## Abstract

Experimental evidence of the interactions among mammalian predators that eat or compete with one another is rare, due to the ethical and logistical challenges of managing wild populations in a controlled and replicated way. Here, we report on the opportunistic use of a replicated and controlled culling experiment (the Randomised Badger Culling Trial) to investigate the relationship between two sympatric predators: European badgers *Meles meles* and western European hedgehogs *Erinaceus europaeus*. In areas of preferred habitat (amenity grassland), counts of hedgehogs more than doubled over a 5-year period from the start of badger culling (from 0.9 ha^−1^ pre-cull to 2.4 ha^−1^ post-cull), whereas hedgehog counts did not change where there was no badger culling (0.3–0.3 hedgehogs ha^−1^). This trial provides experimental evidence for mesopredator release as an outcome of management of a top predator.

## Introduction

Top predators may have far reaching impacts on the ecosystems they inhabit [1], [2]. As a consequence, anthropogenic activities which reduce or remove top predator species may have major and often unintentional effects on the structure, productivity or diversity of the wider ecosystem [1], [3]. In particular, top predators may suppress smaller mesopredators, either by direct intraguild killing or predation or via changes in behaviour such that the smaller predators avoid locations or habitats utilised by the top predator [1], [4]. A decline in the abundance of a top predator may therefore lead to an increase in abundance and/or apparent abundance via ‘mesopredator release’ [5]–[7], sometimes extending further to greater predation pressure on lower trophic levels [5], [8]–[10]. For example, in southern California, Coyotes *Canis latrans* suppress meso-predators (Gray foxes *Urocyon cinereoargenteus,* Striped skunks *Mephitis mephitis* and domestic cats *Felis catus*) such that in habitat patches where coyotes are rare or absent, mesopredator abundance is higher, resulting in the decline of scrub breeding birds [5]. Top predators and mesopredators may therefore interact to shape community structure in a wide range of ecosystems, with important implications for both predator and ecosystem management [1].

There is a growing body of research, which identifies interactions between apex predators and mesopredators that are consistent with the mesopredator release hypothesis [1], [6]. However, the majority of studies have not provided experimental measures of how changes in the abundance of top predators result in changes in mesopredator abundance, but rather describe interactions or associations between species [1], [6]. The paucity of field data relates in part to the logistical and ethical problems associated with accurately estimating and manipulating predator populations [11].

In this study we investigated the relationship between a top predator, the European badger *Meles meles* and a sympatric mesopredator, the western European hedgehog *Erinaceus europaeus,* in the UK. The European badger is a medium-sized mustelid carnivore and has become an apex predator in parts of its range, due to the extirpation of larger terrestrial carnivores [12]. Badgers have a broad omnivorous diet, primarily consisting of invertebrates and plant matter [12], though they also eat smaller mammals including hedgehogs. Hedgehogs are themselves mesopredators predating upon invertebrates, small mammals, reptiles, amphibians and the eggs of ground nesting birds [13], [14]. Previous surveys and manipulations of hedgehog abundance indicate that food availability and badger predation play key roles in determining the abundance, distribution and behaviour of hedgehogs [15]–[18]. Badgers and hedgehogs are not only predator and prey, but also share many of the same food resources and have therefore been considered to interact via intraguild predation, as well as competing for food [18]. Thus there is the potential for badgers to exert a strong influence on hedgehog abundance, as the former can be supported at high density through alternative food resources, even as hedgehog numbers decline [19], [20].

The opportunity to experimentally test the effects of a reduction in predator abundance on populations of a competing prey species arose from the Randomised Badger Culling Trial (RBCT) which was a replicated, controlled field experiment to investigate the effect of culling badgers on the incidence of bovine tuberculosis (TB) in cattle [21]. Previous research has shown that the reduction in badger abundance by culling was associated with increases in the density of red foxes *Vulpes vulpes* [22]. Hence wide-scale badger culling may affect other species that also interact with badgers. We tested the hypothesis that hedgehog abundance and/or behaviour would change, in line with predictions of mesopredator release, as a result of reductions in badger abundance after culling.

## Materials and Methods

### (a) Experimental Design

The design and implementation of the RBCT are fully described elsewhere [23]. Briefly, 10 triplets were established, each consisting of three matched trial areas of approximately 100 km^2^ and which were randomly assigned to proactive badger culling, localized reactive culling following the identification of TB in cattle, or experimental controls with no badger culling. We studied four of the 10 triplets: A (Herefordshire), E (Wiltshire), G (Staffordshire/Derbyshire) and I (Cotswolds). In each triplet, the study ran for 4 to 6 years, including 3 to 5 years of successive annual badger culling (Table 1). For logistical reasons it was not possible to survey for hedgehogs prior to culling in triplets A and E. Hedgehog surveys were also carried out in reactive culling areas before badger culling was implemented but not afterwards and so here they are treated as additional experimental controls.

**Table 1:** The number of years of hedgehog surveys that were carried out in each triplet.

Triplet	Location	Year of initial cull	Number of years ofpre-culling surveys	Number ofyears ofsurveys during culling	Cullingarea	Estimatedreduction inbadger density
**A**	Herefordshire	2000	0	5	A3	32%
**E**	North Wiltshire	2000	0	5	E3	73.2%
**G**	Staffordshire/Derbyshire	2000	1	5	G2	68.8%
**I**	Cotswolds	2002	2	3	I2	39.3%

Estimates of the reduction in badger density are after Smith & Cheeseman (2007).

Within triplets, trial areas exhibited similar densities of badgers prior to the onset of culling [21]. The efficacy of badger culling in the RBCT has been estimated previously by using trapping data [24], [25] and by using signs of badger activity as an index [26]. There was a substantial reduction in badger population in the culled areas compared to experimental control areas in all triplets (Table 1;[25], [26]).

### (b) Data Collection

Hedgehog surveys were carried out annually between July and September (following Doncaster [18]). Within each trial area, 12 fields were selected for survey. Nine pasture fields were selected randomly from all fields available within a 1 km radius of a village and three fields of amenity grassland, which is commonly a preferred habitat for hedgehogs [15], were selected in or on the edge of villages[27].

In each year, fields were surveyed over three separate visits between the hours of 23∶00 and 03∶00 [16], [27]. Each field was systematically searched for hedgehogs using spotlights and hedgehogs were uniquely but temporarily marked [27]. It was assumed that hedgehogs would lose their marks between years. The total number of individual hedgehogs caught at each site over the three repeat visits was taken as an index of relative hedgehog abundance/activity.

### (c) Data Analysis

The count of individual hedgehogs in each field over three visits for a given year was treated as the response variable. To analyse variation in the count of hedgehogs, we fitted a generalised linear mixed model (GLMM) with triplet and treatment as fixed categorical variables and treatment year as a continuous variable. The model was fitted with an Iterative Reweighted Restricted Maximum Likelihood (IRREML) procedure with a negative binomial error structure and a logarithm link function. The area (m^2^) of each field was log-transformed and entered as an offset into the IRREML model, to take account of variability in field size (i.e. survey effort). Treatment had two levels: culled (an area after the initiation of badger culling) or not culled (treatment areas before the initiation of badger culling and experimental control areas with no culling). The term field, nested within triplet and treatment, was entered as a random term.

## Results

**Figure 1:**
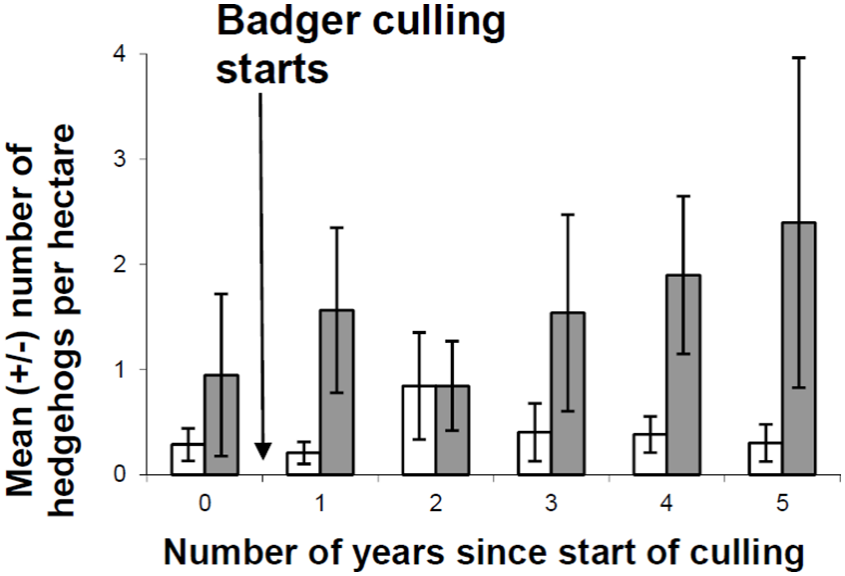
Mean hedgehog density on amenity grassland fields during the Randomised Badger Culling Trial. Shaded columns show badger culling areas and white columns show experimental control areas.

In amenity grassland, there was a significant effect of the interaction between badger culling and the year of culling on hedgehog count (χ^2^ = 8.61, d.f. 1, p = 0.004) (Table 2). No other factors were found to have a significant effect (Table 2). By the end of culling operations, hedgehog counts on amenity grasslands had more than doubled in badger culling areas compared to areas with no culling (Figure 1). Mean hedgehog counts ranged from 0.2–1.0 hedgehog ha^−1^ where badgers were not culled to 0.9–2.4 hedgehogs ha^−1^ where badgers were culled (Figure 1). In pasture fields, only 12 individual hedgehogs were found in 22% of fields and so there were too few observations to carry out statistical analyses.

**Table 2:** Summarised results of GLMM explaining variance in annual counts of hedgehogs on amenity grassland in relation to experimental badger culling.

Sequentially adding terms to fixed model			
Fixed terms	Wald statistic	d.f.	*p*-value
Triplet	2.7	3	0.454
Treatment	2.84	1	0.094
Year	1.6	1	0.208
Treatment×Year	8.61	1	0.004

## Discussion

In line with predictions of the mesopredator release hypothesis, experimental reduction in the badger population resulted in an increase in the count of hedgehogs in amenity grassland habitats. Hedgehog populations and/or behaviour may, therefore, be constrained due to competition and/or predation, or the threat of predation, by a larger predator. This result, suggests that lethal control of badger populations may result in changes to the structure of the wider predator community.

Over the course of this study, the numbers of hedgehogs caught in amenity grassland fields increased by approximately 100% in the areas where badgers were culled, but not in the control areas where culling did not take place. Larger predators may suppress smaller mesopredators either by direct predation/conflict, or by changing their behaviour such that they avoid habitats or locations where the larger predator is present [1], [28]. It is therefore possible that increased captures of hedgehogs in the current study were due not to changes in hedgehog numbers, but to changes in hedgehog behaviour, with hedgehogs being more visible or active in amenity grassland sites where badger numbers had been reduced. Female hedgehogs may avoid larger garden habitats associated with increased badger activity, presumably due to high risk of predation [29]. However, badgers and hedgehogs have also been observed regularly using the same areas [30]. In addition, a concurrent telemetry study of hedgehogs in the Cotswold triplet of the RBCT [31] found no significant effects of badger culling on ranging behaviour that would be consistent with increased counts in amenity grassland sites. This suggests that the increase in hedgehog observations was unlikely to be due to changes in behaviour.

Previous studies indicate that badger predation is one of the main causes of hedgehog mortality [15], [17], [18], and that badger density correlates negatively with hedgehog abundance. It therefore seems likely that the observed increase in the counts of hedgehogs in the current study reflects an increase in hedgehog abundance facilitated by reduced predation and higher survival. This is also consistent with previous research suggesting that badger predation has negative impacts on hedgehog population growth [15].

The analyses in this study were carried out on the numbers of hedgehogs caught on amenity grassland sites, as very few hedgehogs were observed in pasture fields. Amenity grasslands and fields close to villages or houses may be key habitat for hedgehogs and offer a potential refuge against predation by badgers, which are typically less active in these areas, presumably due to human disturbance [16], [17], [27]. Hedgehog presence on amenity grassland shows that prey species can coexist with predators at a landscape scale by occupying areas of habitat that are more favourable to the prey species, perhaps in terms of reduced predation risk or improved food availability [16], [19].

In the context of mesopredator release, interspecific interactions are often viewed as a simplistic three level interaction between top predator, mesopredator and small prey, particularly when the apex predator in question is an obligate carnivore with little dietary overlap with mesopredators [1]. In such circumstances, mesopredator release may increase predation pressure on the species that are preyed upon by the mesopredator, potentially resulting in population declines [5], [8]. The consequences to the wider ecosystem of changes in badger and hedgehog numbers are harder to predict, as both species have broad and largely overlapping dietary niches [12]. The role of omnivores in food web dynamics is not well understood, although they may have stabilising effects by feeding across habitats and trophic levels [32]. Increases in hedgehog numbers may result in increased predation pressure on certain prey species. Hedgehogs may occasionally predate large numbers of single invertebrate and vertebrates species [14]. For example they have been shown to have significant impact on populations of ground nesting birds under certain conditions, via predation of nests [33]. However, it is also possible that a decline in badgers and resultant increase in hedgehogs will have negligible effects on lower trophic levels, either because prey species were already being consumed by badgers, or because specific prey species constitute a small component of hedgehog diets. In conclusion, this study demonstrates that a medium-sized, mustelid omnivore may act to constrain a smaller mesopredator. This study also illustrates the value of field experiments to assess the potential effects of management strategies on the abundance of wildlife populations. European badgers are a wildlife reservoir for bovine tuberculosis in the UK and Ireland and are consequently of intense management interest [26]. This study provides information for assessing the potential ecological consequences of badger culling and further confirmation that a reduction in badger numbers will have direct impacts on other mammal species [22].
